# When Artificial Intelligence Voices Human Concerns: The Paradoxical Effects of AI Voice on Climate Risk Perception and Pro-Environmental Behavioral Intention

**DOI:** 10.3390/ijerph20043772

**Published:** 2023-02-20

**Authors:** Binbin Ni, Fuzhong Wu, Qing Huang

**Affiliations:** 1 College of Media and International Culture, Zhejiang University, Hangzhou 310058, China; 2 School of Journalism and Communication, Tsinghua University, Beijing 100084, China

**Keywords:** AI voice, perceived identity oneness, auditory fear, risk perception, pro-environmental behavioral intention

## Abstract

Artificial intelligence (AI)-enabled text-to-speech transformation has been widely employed to deliver online information in various fields. However, few studies have investigated the effect of the AI voice in environmental risk communication, especially in the field of climate change, an issue that poses a severe threat to global public health. To address this gap, the current study examines how the AI voice impacts the persuasive outcome of climate-related information and the potential mechanism that underlies this process. Based on the social and affect heuristics of voice, we propose a serial mediation model to test the effect of climate-related information delivered by different voice types (AI voice vs. human voice) in eliciting risk perception and motivating pro-environmental behavioral intention. Through an online auditory experiment (N = 397), we found the following. First, the AI voice was as effective as the human voice in eliciting risk perception and motivating pro-environmental behavioral intention. Second, compared with human voice, the AI voice yielded a listener’s lower level of perceived identity oneness with the speaker, which decreased risk perception and subsequently inhibited pro-environmental behavioral intention. Third, compared with human voice, the AI voice produced a higher level of auditory fear, which increased risk perception and thereby led to stronger pro-environmental behavioral intention. The paradoxical role of the AI voice and its wise use in environmental risk communication for promoting global public health are discussed.

## 1. Introduction

The Intergovernmental Panel on Climate Change (IPCC) has recently published a report “Climate Change 2021: The Physical Science Basis” (IPCC, 2021), predicting that human societies would face severe consequences related to climate change in the following decades if no effective countermeasures were taken. However, the global public have been cynical and apathetic about climate issues. For instance, only 18% of respondents from China regarded climate change as a very severe problem (Pew Research Center, 2015). A US-based survey in 2021 showed that 16% of Americans did not believe climate change was occurring, and 33% of them denied that human activities had caused global warming [1]. Similarly, previous literature has demonstrated that the public attached less importance to climate change than to other environmental risks, such as genetically modified foods and nuclear energy [2]. In fact, climate change has been posing a severe threat not only to the ecological system, but also to the health of the global public [3]. For instance, higher temperatures and lower air qualities have accelerated the spread of infectious diseases. Rising sea levels have inundated lowland coastal populations and resulted in the growing phenomenon of environmental refugees; moreover, mortalities have been rising due to the extreme weather, droughts, and floods caused by climate change every year [4]. In order to mitigate the threats to public health resulted from climate change, governments and news media have initiated a number of campaigns to raise public risk perceptions of climate change, as well as to encourage people’s pro-environmental behaviors to combat global warming.

In persuasive communication, media modality plays an important role in shaping people’s risk perceptions of climate change and the associated pro-environmental behavioral intentions [5]. In addition to the commonly used modalities (e.g., text, picture, audio, video) to communicate climate-related messages [6], increasing information has been delivered through artificial intelligence (AI) technologies [7,8]. Notably, the artificial intelligence (AI) voice has been widely used in various fields, such as information seeking, product purchase, automatic text-to-speech transformation, and news anchoring [9,10,11]. Despite the prevalent use of the AI voice in these fields, it remains unclear that how this emerging technology may influence the persuasive effect of climate-related messages. To address this gap, this study tries to compare the effect of an AI voice and a human voice on public risk perceptions of climate change and their pro-environmental behavioral intentions, as well as to unveil the mechanism underlying the relationship between two types of voices and their communicative outcomes.

As a specific media modality, voice may shape people’s perceptions, judgments, and decisions not only through suggesting the social identities of the speaker [12], but also through the voice’s signaling sensory cues, such as speed and pitch [13]. Accordingly, we argue that individuals tend to perceive an AI voice differently from a human voice, not only basing on the social identity suggested by the speaker, but also according to the voice’s sensory characteristics. Drawing on self-expansion theory and social identity theory, we infer that compared with the human voice, the AI voice tends to attenuate a listener’s perceived identity oneness with the speaker, a perception of self-other identity overlaps [14], which may lower the listener’s risk perception of climate change and pro-environmental behavioral intention. Meanwhile, inspired by the literature on fear appeal in risk communication [15], we speculate that the AI voice as compared with the human voice may activate a listener’s auditory fear, a sensory response toward harsh and uncanny voices, which may amplify the listener’s risk perception and increase his or her pro-environmental behavioral intention.

Taken together, we propose a mediation model in which identity oneness and auditory fear serve as two parallel mediators between voice type (AI voice vs. human voice) and risk perception and pro-environmental behavioral intention. The model is tested through an online experiment. Hopefully, the results of this study would shed light on the role of the AI voice in persuasive risk communication and provide some implications for increasing public risk perceptions of climate change and pro-environmental behavioral intentions with the help of AI-facilitated technologies.

We organize the remaining part of the article as follows. Section 2 reviews the relevant theories and empirical evidence, defines the key concepts, and proposes the research questions and hypotheses. Section 3 describes the research methods, participants recruitment, and analytical strategies. The results of the data analysis are presented in Section 4, and the theoretical contributions and practical implications are discussed in Section 5. Section 6 summarizes the main findings and contributions of this study.

## 2. Theoretical Background and Hypotheses Development

### 2.1. Persuasive Effects of AI Voice and Human Voice 

To our knowledge, no study has directly compared the persuasive effect of the AI voice with human voice in risk communication. However, research related to human-computer interaction (HCI) has provided some insights into understanding this difference. On the one hand, researchers argued that compared with a human voice, an AI voice would be less fluent, natural, and credible, which tended to lower the persuasive effect [16,17,18,19]. This drawback of the AI voice was also documented in consumer behavior research, in which consumers exposed to a human voice exhibited stronger purchase intentions than those who interacted with an AI voice [20]. On the other hand, recent studies have challenged this stereotype of the AI voice, suggesting that it is not necessarily less effective than human voice in persuasion. For instance, the AI voice and human voice were equally effective in influencing listeners’ trust in the speaker and enhancing their positive attitudes toward university-wide comprehensive exams [21]. Moreover, individuals with high levels of openness to experience were more likely to be persuaded by robots than by humans in an advertising video [22].

Given the inconsistent findings, we argue that the persuasive effect of an AI voice, as compared to a human voice, may be conditional on various factors such as persuasive contexts and message topics. No study, however, to our knowledge, has compared how an AI voice differs from a human voice in changing people’s perceptions of or behavioral intentions toward environmental risk issues. Therefore, this study aims to address this research gap by comparing the persuasive effect of the AI voice with that of the human voice in communicating climate change issues. Despite those multiple indicators that were used to measure the persuasive effect of a climate-related message (e.g., policy support, donating intention, climate change beliefs, attitudes towards climate change) [23,24,25,26], abundant studies focused on two indicators: (1) risk perception, namely, the extent to which one perceives the threats of climate change as severe; and (2) pro-environmental behavioral intention, that is, the extent to which one is willing to take pro-environmental actions to combat climate change [27,28]. Notably, risk perception and pro-environmental behavioral intention may function as two critical antecedents that predict actual pro-environmental actions [29]. Thus, we ask the following questions:

**RQ1:**  Compared with the human voice, does the AI voice have a stronger direct effect on a listener’s risk perception?

**RQ2:**  Compared with the human voice, does the AI voice have a stronger direct effect on a listener’s pro-environmental behavioral intention?

*Moreover, the underlying mechanism of the AI voice’s persuasive effect, as compared with that of the human voice, has not been fully addressed. Given the distinctions between these two types of voices in various aspects, the AI voice and human voice may both elicit attitudinal change and behavioral intention, but via different approaches. Thus, we propose two additional questions to explore the indirect effects of an AI voice and human voice on listeners’ risk perception and pro-environmental behavioral intentions toward climate change:*

**RQ3.1:**  What are the mechanisms underlying the effect of voice type (AI voice vs. human voice) on risk perception?

**RQ3.2:**  What are the mechanisms underlying the effect of voice type (AI voice vs. human voice) on pro-environmental behavioral intention?

### 2.2. Social Heuristics: The Mediating Role of Perceived Identity Oneness

One of the most obvious differences one could detect between an AI voice and a human voice is the identity of the speaker. In relation to this, a listener’s perceived identity oneness with a speaker may influence the persuasive outcomes. Perceived identity oneness describes the extent to which an individual perceives an identity overlap with certain social group members [14,30]. This concept is developed from the self-expansion theory, which suggests that people have an initial need for connectedness and may develop relational and collective self-construal by integrating others of certain social groups into their self-concept [31]. Thus, the sense of identity oneness appears not only in close relationships (e.g., partners, friends) [32,33], but also between in-group members and cross-group members [34,35], which depends on how an individual feels psychologically close to another and perceives the similarities between them. As supported by the social identity theory, individuals are typically more psychologically merged with the social group that they identify with, and thus perceive a high level of identity oneness with in-group members than with out-group members [33,36].

Drawing on the Computers Are Social Actors (CASA) hypothesis [37], people tend to apply norms and scripts of human-to-human interactions, including the unconscious tendency of social categorization and intergroup differentiation, to interactions with AI agents [38,39]. Evidence has shown that people refer to robots as social “others” and may resist working with them because they pose an identity threat to human beings [40,41]. Likewise, when interacting with robots that are less human-like, individuals would feel a stronger sense of alienation, attach more prejudice, and exhibit a less positive attitude toward them [42,43]. Although AI’s degree of anthropomorphism has largely been improved in recent years, people tend to interact with AI agents differently from real humans in various contexts, such as online learning, medical service, and ethical decisions [43,44,45]. Thus, people may categorize AI agents as out-group members, as compared with human agents as in-groups members, and feel less at one with AI agents than with humans, especially when they detect conflicts or disparities between robots and humans [43,46].

This intergroup differentiation is also manifested and even amplified in auditory communication. On the one hand, voice carries rich information about the social identity of the speaker (e.g., race, gender, nationality), which activates listeners’ stereotypes about the imagined speaker [47,48,49]. Accordingly, listeners quickly recognize the speaker as an out-group member when they hear an AI voice, which prevents listeners from developing identity oneness with the AI speaker. On the other hand, the social distance between listeners and the speaker is further increased by the speaker’s auditory characteristics. Specifically, AI-synthesized voice has been criticized as unnatural and dehumanized with obvious machinery cues [50,51]. Thus, an AI voice, as compared with a human voice, might be less effective in building emotional and relational bonds between the listener and the speaker, thereby inhibiting the listener’s perceived identity oneness with the speaker. Hence, we put forward the following hypothesis:

**H1:**  *Compared with human voice, AI voice leads to a listener’s lower level of perceived identity oneness with a speaker.*

Moreover, identity oneness between a listener and a speaker improves persuasive effects. According to Cialdini et al. (1997) [14], individuals who experience a high level of oneness with another one may feel psychologically closed to this person and are motivated to take his or her perspectives. This has been documented in abundant studies that examined the identity match effect regarding partisanship, race, and gender in persuasion.

For example, people endorsed the message delivered by a speaker from their own political party, and exhibited ideological rejection toward messages from the rival party, given that common identity bred shared values [52]. In a similar vein, white participants evaluated white speakers more positively and were more likely to be persuaded to engage in the movement supporting the black’s rights, as compared with a black or anonymous speaker [53]. Furthermore, people evaluated messages from the same gender as more credible and trustworthy than those from another gender [54].

The identity match effect also pertains to risk issues. When exposed to a narrative about gender violence, participants reported a high level of risk perception of gender violence when the victim was portrayed as from the same country as the participants, compared with the victim from another country. Particularly, feeling oneness with the information source functions as a critical predictor of the audience’s attitudinal change about climate policies [55]. In this study, listeners’ perceived identity oneness with the speaker tends to increase their risk perceptions of climate change and facilitate their pro-environmental behavioral intentions, which is stated in the following hypothesis:

**H2:**  *Perceived identity oneness is positively associated with risk perception (H2.1) and pro-environmental behavioral intention (H2.2).*

Taken together, H1 and H2 suggest that perceived identity oneness might serve as a mediator between AI voice and persuasive outcomes, which is formulated as another hypothesis:

**H3:**  *Perceived identity oneness negatively mediates the effect of AI voice on risk perception (H3.1) and pro-environmental behavioral intention (H3.2).*

### 2.3. Affect Heuristics: The Mediating Role of Auditory Fear

Fear describes an unpleasant state when people feel uncertainty and lack of control in the environment, and is characterized by heightened autonomic activities involving the nervous system [56]. Fear can be elicited through listening to certain sounds and functions as a sensory and emotional response [57]. We term this voice-induced negative experience as auditory fear.

Widely used AI voices, especially those with a low audio sample rate and a poor quality, are obvious of digital manipulation, which may leave listeners’ with unnatural, rough, and even harsh feelings [58,59]. When exposed to a noisy voice, people tend to feel nervous, tense, stressful, and anxious [60,61]. This may also pertain to people’s responses to the imperfect AI voice. Although sophisticated synthetic techniques have been applied to improve the AI voice, people still feel uncomfortable with it. This may be because the AI voice is created through synthesizing human speeches without fully integrating the tones, intonations, and emotions of human expressions, which makes listeners feel uncertain about whether the speaker is animate or inanimate [62].

Several studies have supported that an AI voice is likely to trigger listeners’ uncomfortable feelings. For instance, in a qualitative investigation, participants reported feeling nervous when listening to the creepy, disturbing, and weird AI voice [63]. Moreover, evidence has shown that the less a synthetic speech is human-like, the more unpleasant it tends to elicit [64,65]. Similarly, people evaluated the virtual character as more unfriendly and uncanny when it was paired with an AI voice than with a human voice [66]. As noted above, human listeners consider an AI speaker an out-group member and believe that it poses an identity threat to human beings. Thus, listening to the AI voice is expected to evoke people’s fear of being threatened by the AI out-group members, coupled with feelings of uncertainty and uncontrollability. Taken together, we hypothesize that:

**H4:**  *Compared with human voice, AI voice elicits a listener’s stronger auditory fear.*

According to the appraisal tendency framework, incidental emotions can carry over from past situations to color irrelevant judgements and decisions. This effect occurs when the core appraisal dimensions of the emotion match the salient cognitive dimensions of the judgment at hand [67,68]. For instance, people who are delighted by music may form favorable impressions of others, where the emotion and the subsequent cognition both involve appraisals of pleasantness. Similarly, incidentally induced fear can influence risk assessment, where the emotion and the subsequent cognition both involve appraisals of certainty and control [69].

An appeal to fear serves as an affective heuristic that improves the outcomes of persuasive risk communication. Fear is a critical self-protective mechanism that alerts human beings of the potential dangers and protects us from life-threatening situations [70]. People who feel intense fear tend to pay a close attention to the threat and engage in systematic processing of risk information [71,72,73], which facilitates their elaboration of the relevant evidence and increases their intention to follow the risk-reduction advice suggested by information. Furthermore, according to the protection motivation theory, people are motivated to follow the advice aiming at reducing the surrounding threat to alleviate their fear [74]. Thus, fear-arousing messages increase people’s risk perceptions and enhance their attitudinal and behavioral compliance with the advice recommended by persuasive messages [75,76].

As to climate issues, practitioners have initiated a number of campaigns to raise the public’s awareness of the catastrophic consequences of global warming [77]. This fear appeal has also been demonstrated to enhance people’s climate risk perceptions and pro-environmental behavioral intentions [75,78]. Empirical studies have shown that people tend to make a pessimistic risk assessment and prefer risk-averse decisions when they feel intense fear [75,79,80]. Therefore, when listening to climate risks-related messages broadcasted by an AI voice, listeners’ auditory fear may influence their assessment about climate risks, leading them to focus on the severe consequences of climate change, which then encourages their preventive behaviors. Thus, we put forward the following hypotheses:

**H5:**  *Auditory fear is positively associated with risk perception (H5.1) and pro-environmental behavioral intention (H5.2).*

**H6:**  *Auditory fear negatively mediates the effect of AI voice on risk perception (H6.1) and pro-environmental behavioral intention (H6.2).*

### 2.4. Parallel Mediation Effect of Perceived Identity Oneness and Auditory Fear

Risk perception is a powerful predictor of pro-environment behavioral intention. When people realize the risks induced by environmental problems, they tend to take these problems seriously, care about the mitigative measures, and are motivated to act pro-environmentally [81,82]. Abundant studies have demonstrated that people’s risk perceptions of environmental problems in general and in specific domains (e.g., air pollution, marine plastic pollution, and climate change) directly and positively predict their pro-environmental attitudes and behaviors [29,83,84]. More importantly, empirical evidence has shown that people are more inclined to adopt pro-environmental behaviors because they perceive a high level of threat or worry about climate change [85,86]. Thus, we hypothesize that:

**H7:**  *Risk perception is positively associated with pro-environmental behavioral intention.*

According to the stimulus-organism-response (SOR) model, information from the environment (i.e., stimulus) induces cognitive and affective changes in the receiver (i.e., organism), which in turn, leads to behavioral changes (i.e., response) [87]. The SOR model has been widely used to explain individuals’ pro-environmental behaviors [87,88,89]. In this study, risk information delivered by different voices—AI voice and human voice—results in changes in listeners’ perceived identity oneness and auditory fear, which in turn stimulates their pro-environmental behavioral intentions. Accordingly, we posit two serial mediation paths from AI voice, as compared with human voice, to pro-environmental behavioral intention:

**H8:**  *Perceived identity oneness and risk perception serially mediate the relationship between AI voice and pro-environmental behavioral intention*.

**H9:**  *Auditory fear and risk perception serially mediate the relationship between AI voice and pro-environmental behavioral intention*.

**Figure 1:**
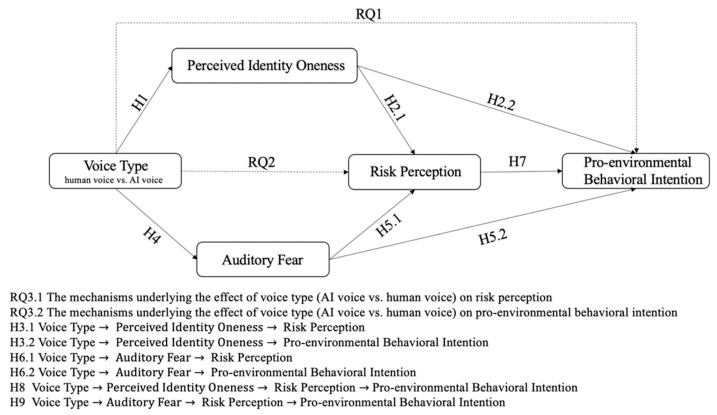
The hypothesized model.

Figure 1 demonstrates the overall hypothesized model of the current study.

## 3. Materials and Methods

### 3.1. Participants

Studies have shown that online auditory experiments are as valid as laboratory and field experiments and can be conducted more effectively [90]. Thus, we conducted an online experiment to test the hypothesized model. Participants were recruited using the sample service provided by Sojump (http://www.sojump.com, accessed on 30 March 2022), a professional online survey platform with a sample pool of 6.2 million registered users with diverse demographic characteristics in mainland China. Users were invited to join the sample pool once they have completed online surveys using Sojump. After providing demographic information (e.g., age, gender, occupation, income), potential participants would routinely receive survey tasks through emails or from the website. This platform has been widely employed by previous studies to conduct online experiments [90,91,92]. Since our online experiment involved auditory stimuli, participants were asked whether they stayed in a quiet environment that allowed them to play and listen to the audio (see Audio S1) clearly. Only those who indicated yes proceeded to the formal experiment.

Four hundred and thirty-nine participants completed the online experiment. We excluded participants (1) who submitted multiple replies using the same IP address or (2) those who did not pass the attention checks that either tested whether they listened to the audio (see Audio S1) carefully or asked them to choose a certain option. Finally, a total of 397 valid participants were included in the formal analysis. Table 1 displays the demographic characteristics of the sample.

### 3.2. Stimuli

To increase the ecological validity of the experiment, a news article about climate change published in China News, a widely used news mobile application in China, was chosen as the experimental stimuli. This application provides a “text-to-speech” option such that users can listen to the news articles delivered by an AI voice by simply clicking the button on the interface. An explanatory article about the effects of climate change was chosen to ensure that the content was concise, accurate, and easy to understand (The news article was entitled “Climate change is relevant to you and me! Foreign media anticipated the impacts of global warming”. It was published on 19 July 2021, https://baijiahao.baidu.com/s?id=1705703090080832215&wfr=spider&for=pc, accessed on 5 March 2022). To increase participants’ attention span to the experimental stimuli, we only kept the paragraphs that described climate change risks and encouraged people to take action, and thus reduced the length of the news article to 274 Chinese words. The detailed news text used as the experimental stimuli is shown in Table 2.

Next, we transformed this news article into audios. The AI voice was created using the AI system embedded in China News mobile application. Given that the vast majority of intelligent voice assistants use female voices by default, such as Amazon’s Alexa, Microsoft’s Cortana, Google’s google assistant, etc. Moreover, compared to the voice of a male robot, most people prefer the voice of a female robot [93]. Thus, we employed a female AI voice to broadcast. The human voice was recorded by a professional female news anchor.

### 3.3. Procedure

After signing the consent form to participant in the experiment, participants were randomly assigned to either of the two conditions. In the experimental condition (N = 205), participants listened to the auditory news delivered by the AI voice. In the control condition (N = 192), participants listened to identical auditory news delivered by the human voice. To ensure that participants completed listening to all the contents, a timer was set on the web page that presented the stimuli. After exposure to the stimuli, participants were asked to report perceived identity oneness, auditory fear, risk perception, and pro-environmental behavioral intention, as well as demographic variables (gender, education, monthly income, and age).

### 3.4. Measures

#### 3.4.1. Manipulation Check

Before listening to the audio (see Audio S2) of the experiment, we informed the participants of the news source and the identity of the speaker (i.e., a human speaker or an AI speaker). After completing all experiments, participants were asked: “Do you think the speaker you just listened to be a real person or an artificial intelligence?”. This binary item was used to ensure that participants had accurately recognized the identity of the speaker. All participants passed the manipulation check.

#### 3.4.2. Perceived Identity Oneness

**Figure 2:**
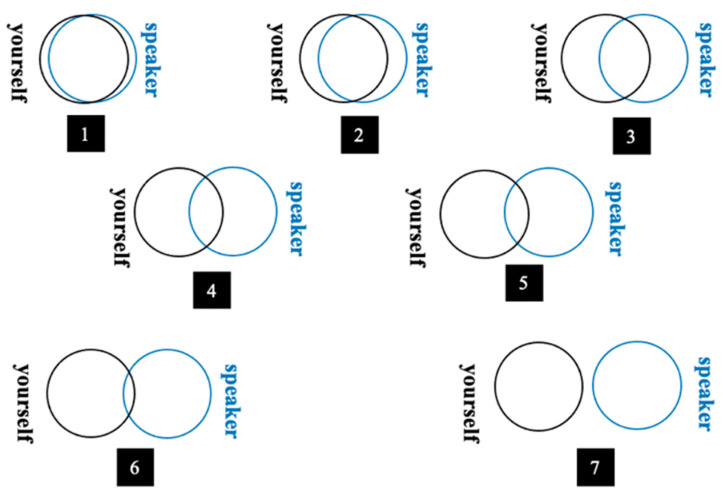
Measurement of perceived identity oneness. *Note.* black circle: yourself, blue circle: the speaker.

Perceived identity oneness was measured by the inclusion of other in the self (IOS) scale developed by Aron et al. (1992). The IOS scale employed a single-item pictorial measure to tap directly one’s perceived identity overlap and interpersonal closeness with others [94]. Participants were asked to select the picture that best described their relationship with the speaker from seven Venn-like diagrams, with each representing different degrees of overlap of two circles. As shown in Figure 2, the more the two circles detached from each other, the lower identity oneness between the speaker and the listener was. Thus, a higher IOS score represented a lower level of perceived identity oneness. Responses were reverse coded to reflect perceived identity oneness (M = 4.60, SD = 1.52).

#### 3.4.3. Auditory Fear

Auditory fear was measured by a 4-item scale based on the previous measurement of fear in risk communication [95]. The original scale was adapted to capture the participants’ fearful feelings triggered by voice, rather than by the texts. Participants were asked to rate from 1 (not at all) to 7 (very much) to indicate the extent to which the *voice* they heard made them feel (1) nervous, (2) fearful, (3) vigilant, and (4) afraid. The four items were averaged to create an index of auditory fear (M = 3.95, SD = 1.33; Cronbach’s α = 0.859).

#### 3.4.4. Risk Perception

The 6-item scale of risk perception was adapted from Ogunbode et al. (2020) to measure one’s perceived severity of the negative consequences of climate change [96]. Participants were asked to indicate the degree of severity in the following aspects (not at all = 0, extremely severe = 100): the threat that climate change poses to (1) you, (2) your country, (3) the humanity, or (4) the whole world; and the negative consequences of climate change would cause (5) in the following few years, or (6) in the next 100 years. The six items were averaged to index risk perception (M = 75.66, SD = 13.46; Cronbach’s α = 0.860).

#### 3.4.5. Pro-Environmental Behavioral Intention

The instrument of pro-environmental behavioral intention was adapted from Tsai et al. (2021) [97]. Participants rated on a 5-point Likert scale (1 = “not at all”, 5 = “very willing”) to indicate the extent to which they were willing to take the following actions to combat climate change. Sample items included: “I am willing to reduce the use of plastic products (e.g., plastic bags)”; “I am willing to pay a higher price for environmentally friendly products”; and “I am willing to spend time and effort to persuade others to save energy.” The 12 items were averaged to index pro-environmental behavioral intention (M = 4.06, SD = 0.47; Cronbach’s α = 0.812).

### 3.5. Statistical Analyses

We used SPSS version 26.0 to conduct all the statistical analyses. First, we performed two analyses of variance (ANOVAs) to examine the main effects of voice type on risk perception and pro-environmental behavioral intention, respectively. Next, we used PROCESS [97] based on a bootstrap sample of 5000 with 95% bias-corrected confidence intervals to test the mediating effects. Specifically, the parallel mediating roles of perceived identity oneness and auditory fear were tested using PROCESS model 4. The serial mediating effects were tested using PROCESS model 80. (Two serial mediating effects were (1) voice type → perceived identity oneness → risk perception → pro-environmental behavioral intention; and (2) voice type → auditory fear → risk perception → pro-environmental behavioral intention.) The mediating effects were significant if the confidence interval did not include zero. Standardized coefficients were reported.

## 4. Results

### 4.1. Preliminary Analyses 

Results of t-tests revealed no significant differences in gender (t = −0.009, *p* = 0.985), education level (t = 0.335, *p* = 0.424), monthly income (t = −0.271, *p* = 0.467), or age (t = −1.107, *p* = 0.221) between the two conditions. This suggested that the randomization was successful.

### 4.2. Main Effects

To answer RQ1, results of an ANOVA test showed that the main effect of voice type on risk perception was not significant (F [1,395] = 0.088, *p* = 0.767). Specifically, the AI voice (M = 455.14, SD = 81.32) and the human voice (M = 452.72, SD = 80.37) was equally effective in eliciting risk perception.

To answer RQ2, another ANOVA test demonstrated the main effect of voice type on pro-environmental behavioral intention was not significant (F [1,395] = 0.275, *p* = 0.600). Specifically, the AI voice (M = 48.81, SD = 5.24) and the human voice (M = 48.52, SD = 6.11) was equally effective in inducing pro-environmental behavioral intention.

### 4.3. Parallel Mediation Effects

We conducted several mediation analyses to answer RQ3.

The parallel mediating effects of perceived identity oneness and auditory fear in the relationship between voice type (0 = human voice, 1 = AI voice) and risk perception were analyzed using PROCESS Model 4. Results showed that the AI voice, as compared with the human voice, led to a lower level of perceived identity oneness with the speaker (β = −0.32, *p* < 0.01), supporting H1. Perceived identity oneness positively predicted risk perception (β = 0.22, *p* < 0.001), supporting H2.1. Furthermore, perceived identity oneness negatively (β = −0.07, 95% CI [−0.14, −0.03]) mediated the effect of the AI voice on risk perception, supporting H3.1.

Similarly, the AI voice, as compared with the human voice, led to a higher level of auditory fear (β = 0.43, *p* < 0.001), showing support for H4. Auditory fear positively predicted risk perception (β = 0.15, *p* = 0.003), which supported H5.1. Moreover, auditory fear positively (β = 0.07, 95% CI [0.02, 0.14]) mediated the effect of the AI voice on risk perception, supporting H6.1. Model 1–3 in Table 3 display the detailed regression results.

The parallel mediating effects of perceived identity oneness and auditory fear in the relationship between voice type (0 = human voice, 1 = AI voice) and pro-environmental behavioral intention were analyzed using PROCESS Model 4. As aforementioned, the AI voice, as compared with the human voice, led to a lower level of perceived identity oneness with the speaker (β = −0.32, *p* < 0.01). Meanwhile, perceived identity oneness positively predicted pro-environmental behavioral intention (β = 0.22, *p* < 0.001), showing support for H2.2. Taken together, perceived identity oneness negatively mediated the effect of AI voice on pro-environmental behavioral intention (β = −0.07, 95% CI [−0.14, −0.03]), supporting H3.2.

As mentioned earlier, the AI voice tended to lead to a higher level of auditory fear than human voice did (β = 0.43, *p* < 0.001). Additionally, the association between auditory fear and pro-environmental behavioral intention was not significant (β = 0.001, *p* = 0.991). Thus, auditory fear did not significantly mediate the relationship between voice type and pro-environmental behavioral intention (β = 0.0003, 95% CI [−0.04, 0.04]. Hence, H5.2 and H6.2 was not supported. Model 1, 2, and 4 in Table 3 describes the detailed regression results.

### 4.4. Serial Mediation Analyses

We further answer RQ3 by verifying two serial mediation effects.

The serial mediating effects were tested using PROCESS model 80. Results indicated that risk perception positively predicted pro-environmental behavioral intention (β = 0.29, *p* < 0.001), supporting H7. The AI voice, as compared to the human voice, yielded a lower level of perceived identity oneness, which then resulted in a lower level of risk perception, which in turn, led to a weaker pro-environmental behavioral intention (β = −0.02, 95% CI [−0.04, −0.01]). Thus, H8 was supported. Additionally, the AI voice, as compared to the human voice, produced a higher level of auditory fear, which positively predicted risk perception, which in turn, led to a stronger pro-environmental behavioral intention (β = 0.02, 95% CI [0.01, 0.04]). Thus, H9 was supported. Model 1–5 in Table 2 demonstrates the regression results.

**Figure 3:**
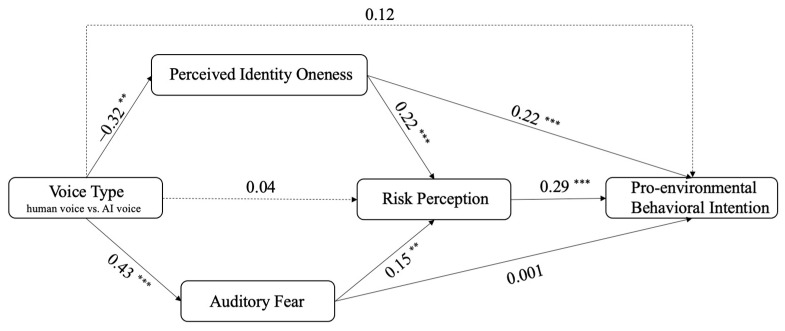
The final model based on statistical results. *Note.* N = 397. Voice type: human voice = 0, AI voice = 1; ** *p* < 0.01, *** *p* < 0.001.

Figure 3 presents the final model with statistical results.

## 5. Discussion

This study compared the effect of the AI voice with that of the human voice in eliciting climate risk perception and pro-environmental behavioral intention and illustrated the underlying mechanism between these variables. Through an online experiment, we found that the AI voice was as effective as the human voice in triggering climate risk perception and pro-environmental behavioral intention. Mediation analyses further revealed the paradoxical role of the AI voice in influencing climate risk persuasion. On the one hand, the AI voice yielded a listener’s low level of perceived identity oneness with the speaker, which resulted in decreased persuasive effect, namely, a low level of risk perception and the subsequent pro-environmental behavioral intention. On the other hand, the AI voice induced a high level of auditory fear, which led to increased persuasive effect, namely, a high level of risk perception and the subsequent pro-environmental behavioral intention. All hypotheses were supported except for H5.2 and H6.2. Specifically, auditory fear did not show a significant direct effect on pro-environmental behavioral intention but demonstrated an indirect effect on it via the mediating role of risk perception. We speculated this may due to that auditory fear may also lead to lower efficacy [98], which would also inhibit the listeners’ intention to take actions.

### 5.1. Theoretical Implications

First of all, despite the wide application of the AI voice in media industries, few studies have investigated the role of AI voice in persuasion, especially in environmental risk communication. To our knowledge, this study first attempts to use the AI-mediated auditory communication research to explicate the persuasive effects of climate-related information. Despite the prevalent concern that the AI voice, as compared with the human voice, may reduce the persuasive effect due to the unnatural and even unpleasant sounds produced by some immature AI voices [51,99], our findings demonstrate that the AI voice is not necessarily less effective than the human voice in persuasion. Moreover, although some studies have exhibited inconsistent findings regarding the effect of AI voices on people’s attitudinal or behavioral changes [99,100,101], we argue that the persuasive effect of the AI voice depends largely on the issue under examination.

Second, we extend the research on voice type in persuasion by unraveling the mediating mechanism underlying the effect of the AI voice and human voice on persuasive outcomes. Our results demonstrate that the mediating mechanism consists of a positive and a negative indirect path, which suggests that the AI voice and human voice may lead to persuasive outcomes through triggering different heuristics. We illustrate this in detail as follows.

On the one hand, perceived identity oneness negatively mediated the effect of AI voice on risk perception and pro-environmental behavioral intention. The negative association between the AI voice and perceived identity oneness denotes that people tend to categorize AI broadcasters as out-group members, even though they exhibit the affordance of intimate companions in everyday life [102,103]. This process of intergroup differentiation is easily triggered by listening to the auditory information, echoing the idea that the voice is an important form of social presence and is sufficient to convey the social identity of a speaker [104]. These findings also extend the scope of the self-expansion theory that has been previously used to investigate interpersonal intimacy [31,105,106] to a human-computer interaction context. Moreover, the positive association between perceived identity oneness and persuasive outcomes is consistent with the previous research that people are susceptible to the information delivered by those who share the in-group identity [107], which highlights those social heuristics matter both in interpersonal and AI-mediated risk communication.

On the other hand, auditory fear positively mediated the effect of the AI voice on risk perception and pro-environmental behavioral intention. Although qualitative studies have implied the role of the AI voice in eliciting listeners’ uncomfortable feelings [108], our study bridges the research gap by experimentally testing this effect. Despite that the AI voice can elicit fear-related emotions when it is delivered by a broadcaster with high human realism [109], our results suggest that the AI voice itself is capable of leaving listeners with feelings such as eeriness, nervousness, and fear. Furthermore, the positive association between auditory fear and risk perception corroborates that fear appeals promote the persuasive effect of risk information. Specifically, our study demonstrates that feelings of fear can be elicited by certain characteristics of media modality, such as voice, to improve the persuasive effectiveness. This finding provides a novel insight into understanding the role of AI-induced negative emotions in persuasive risk communication. AI voices are not necessarily an inhibitor of persuasion due to their unnaturalness and eeriness [63,110]. Instead, these uncomfortable feelings generated by AI voices may be creatively transformed into useful cues to change listeners’ perception and attitude.

### 5.2. Practical Implications

The paradoxical effect of the AI voice, as compared with the human voice suggests that risk communicators, platform developers, and voice designers may utilize these two types of voices to fulfil their strengths whilst circumvent their weaknesses in climate risk communication. First, considering that the AI voice is as effective as the human voice in eliciting risk perception of climate change, which consequently leads to pro-environmental behavioral intention, we advise that risk communicators may consider using the AI voice as a substitute for the human voice in communicating climate risks. The low-cost and convenient speech-to-text transformation functions may further help disseminate the auditory information related to climate change to a broader audience and raise a wider concern about global public health among them.

Second, because the human voice leads to desirable persuasive outcomes of climate-related information through creating a sense of identity oneness, human speakers are suggested to explicitly display their identities and express their attitudes toward climate issues from the standpoint of human beings. Given that human speakers are less effective in eliciting auditory fear than AI voices do, which may inhibit the persuasive effect of risk information, we put forward a few measures regarding voice design to minimize this undesirable effect. For instance, human speakers could present the severe risks of global warming using a tone with a higher pitch and a more serious voice, which may help increase listeners’ fear toward the negative consequences of climate change.

Third, given that the AI voice may lower the persuasive effectiveness of climate-related information due to a lack of identity overlap with human beings, AI designers are advised to build and strengthen the identity connection between an AI speaker and its listeners. This can be completed through improving the anthropomorphism of the AI speaker [111], such as giving the AI speaker a human name, allowing the AI speaker to introduce himself or herself, improving the naturalness of the AI voice, etc. Considering that the AI voice may increase climate risk perception through inducing auditory fear, we suggest that unnatural and eerie AI voices be properly utilized to convey information that highlights the severe consequences of global warming, thus transforming these flawed auditory properties into persuasive advantages. Notably, we are not suggesting maximizing the auditory fear induced by AI voices, since moderate fear, rather than extreme fear, motivates pro-environmental behavioral intentions.

### 5.3. Limitations and Future Research

First, this study employed an online auditory experiment to answer the research questions and test the hypotheses. Although the online experiment helped obtain a relatively large sample than the traditional laboratory experiment did and enhance the ecological validity of the study, we could not fully ensure the participants’ continuous attention toward the auditory stimuli despite the stimuli-related attention checks. Therefore, future studies could replicate the current study through conducting laboratory experiments.

Second, because female AI voices are widely used and preferred than male AI voices, this study investigated the persuasive effect of female AI voices [112]. This may inhibit us from observing whether the persuasive effect can be generalized to male AI voices, and whether the gender of the AI speaker functions as a moderator. Indeed, several studies have suggested gender stereotypes toward AI speakers, that is, male AI voices are perceived as competent and authoritative, while female AI voices are perceived as warm and helpful [113,114]. Future research may further compare male AI voices and male human voices, and whether the persuasive effect of the AI voice, as compared with the human voice, is conditional on the gender of the speaker.

Third, the AI voice used in the experimental stimuli was automatically generated by China News application, which was obvious of machinery cues and may sound unnatural. This might make it easier for the participants to identify the AI voice. However, the use of AI voices with machinery cues could not only enhance the ecological validity of the current study but also contribute to previous studies that investigated the effect of “unnatural” AI voices. With the fast development of AI technologies, more sophisticated techniques are being applied to enhance the fluency, naturalness, and pleasantness of the AI voice, to ensure that the qualities of AI voices are getting closer to human voices. Thus, researchers could employ more natural AI voices as stimuli in the future and investigate whether the main effect and mediation effects in this study hold true. In addition, our study focuses on the persuasive effect of automatically generated AI voices. Furthermore, voice-activated AI assistants that communicate with people have already become commonplace, allowing future research to study the effects of AI voices in interactive contexts.

Fourth, this study provides an initial exploration into the persuasive effect of the AI voice, as compared with the human voice in climate risk communication. Further studies may investigate whether the findings pertain to other contexts of risk communication, such as behavioral preparedness in public health emergencies and new technology adoption. In addition, perceived identity oneness and auditory fear—two mediators in this study—may not fully explain the relative strengths and weakness of AI voices as compared to human voices in risk communication. For instance, future studies may consider other social heuristics (e.g., perceived credibility and perceived warmth) [115,116] and affective heuristics (e.g., hope, worry, and sympathy) as mediators, which have been shown to be critical in climate-related persuasion [25,117].

Lastly, although pro-environmental behavioral intention functions as an antecedent of actual pro-environmental behavior, people do not always act as they intend to do [118]. Thus, future studies may include actual pro-environmental behaviors—such as donating to environmental organizations and reducing carbon footprint—as dependent variables. Moreover, there are multiple factors that may influence one’s actual environmental behavior, such as income, environmental self-identity, cultural values, and social norms [119,120,121]. Future research should consider controlling for these variables to avoid confounding effects or observing whether these variables moderate the effect of voice type on pro-environmental behaviors. More importantly, the experimental design only allows us to observe the immediate effect of information exposure. Thus, future studies should use longitudinal design to investigate how long this effect could last and whether repeated exposure to climate-related information delivered by certain voices may increase pro-environmental behaviors over time through.

## 6. Conclusions

This study compares the persuasive effect of the AI voice with the human voice in the context of climate-related risk communication. While the AI voice exhibits an equal effect as the human voice in eliciting climate risk perception and the subsequent pro-environmental intention, our findings further demonstrate its relative drawback and strength in persuasion. On the one hand, the AI voice, as compared with the human voice, decreases persuasive outcomes, since it is less effective in triggering a listener’s perceived identity oneness with the speaker, which functions as a social heuristic in persuasion. On the other hand, the AI voice, as compared to the human voice, increases persuasive outcomes, since it elicits higher auditory fear, which functions as an affect heuristic in persuasion. By revealing the paradoxical role of the AI voice in climate-related persuasion, this study offers some insights into utilizing AI-mediated risk communication for promoting global public health.

## Figures and Tables

**Table 1:** Demographic characteristics of the sample.

Measure	Item	Frequency	Percentage (%)
Gender	Male	186	46.9%
	Female	211	53.1%
Age	15–18	4	1.0%
	19–24	194	48.9%
	25–34	165	42.5%
	35–44	24	6.1%
	45–65	10	2.5%
Education level	Middle school or below	4	1.0%
	High school	8	2.0%
	Bachelor or vocational school	355	89.4%
	Master or PhD	30	7.6%
Monthly income	Less than 1000 RMB	26	6.5%
	1000–3000 RMB	116	29.2%
	3001–6000 RMB	99	24.9%
	6001–10,000 RMB	115	29.0%
	More than 10,000 RMB	41	10.3%

**Table 2:** Example of experimental stimuli.

News Content: “Climate Change Is Closely Related to You and Me! Foreign Media Anticipated the Impacts of Global Warming.”
Global warming describes the rise of the temperature worldwide resulted from the greenhouse gas effect. Currently, the speed of global warming is accelerating, and is faster than ever before. According to the World Meteorological Organization (WMO), the Earth is nearly 1 °C warmer than it was in the early industrial age. At this rate, the global temperature will be 3 to 5 °C warmer than it was in the pre-industrial age by 2100. Slight as this increase might seem, the Intergovernmental Panel on Climate Change (IPCC) noted that humans would face catastrophic consequences if no effective countermeasures were taken. For instance, the sea levels would rise; some islands and coastal lowlands would be inundated; the temperature and acidity of the seas would increase; agriculture and animal husbandry would face great challenges. Indeed, climate change is relevant to everyone who lives on the planet. Each individual would be affected by global warming if no effective actions were taken. Thus, everyone should contribute to the mitigation of climate change.

**Table 3:** Testing the mediation effects.

Predictors	Model 1	Model 2	Model 3	Model 4	Model 5
	PIO	Auditory Fear	Risk Perception	PEBI	PEBI
	*β* *(SE)*	*β* *(SE)*	*β* *(SE)*	*β* *(SE)*	*β* *(SE)*
Voice Type	−0.32 (0.10) **	0.43 (0.10) ***	0.04 (0.10)	0.12 (0.10)	0.11 (0.10)
PIO			0.22 (0.05) ***	0.22 (0.05) ***	0.16 (0.05) **
Auditory Fear			0.15 (0.05) **	0.001 (0.05)	−0.04 (0.05)
Risk Perception					0.29 (0.05) ***
*R*	0.03	0.05	0.08	0.05	0.12
*F*	10.64 **	19.09 ***	11.34 ***	6.58 ***	13.78 ***

*Note.* N = 397. Voice type: human voice = 0, AI voice = 1; each column represented a regression model, with prediction criteria at the top of the column. Standardized coefficients were reported. PIO = perceived identity oneness. PEBI = pro-environmental behavioral intention. ** *p* < 0.01, *** *p* < 0.001.

## Data Availability

The data are not publicly available due to privacy protection. Please email the corresponding author, Qing Huang, for the data: qing_huang@zju.edu.cn.

## Supplementary Materials

The following supporting information can be downloaded at: https://www.mdpi.com/article/10.3390/ijerph20043772/s1, Audio S1: Experimental stimuli for the human voice condition. Audio S2: Experimental stimuli for the AI voice condition.

## Abbreviations

AI: artificial intelligence. IOS: inclusion of other in the self. PIO: perceived identity oneness. PEBI: pro-environmental behavioral intention.
